# Reliability of Pre-Transplant Live Donor Renal Biopsies in Predicting the Graft Outcome

**Published:** 2014-05-01

**Authors:** G. H. Naderi, M. Sotoudeh, D. Mehraban, S. Nategh

**Affiliations:** 1 *Departments of Urology, Tehran University of Medical Sciences, Tehran, Iran*; 2 *Pathology, Tehran University of Medical Sciences, Tehran, Iran*

**Keywords:** Transplant, Renal biopsies, Graft outcome, Treatment outcome, Kidney, Tissue donors, Creatinine

## Abstract

Background: Biopsy from deceased donors is of great value in predicting the efficacy and mid-term and long-term outcome of kidney transplantation.

Objective: To determine the prevalence of pathological changes in live donors’ kidneys and their association with the graft outcome.

Methods: This cohort study was performed among a group of renal transplant recipients. Biopsy was taken from donor’s kidney. The functionality of the grafted kidney was then evaluated by measuring serum creatinine, based on which, the patients were categorized into “successful” and “unsuccessful” groups. The results were compared based on biopsy results.

Result: We studied 58 kidneys from live donors. The mean±SD urine volume on the first day after transplantation was 10,052±3286 mL. Absence of allograft dysfunction was seen in 55 (95%) patients during a month, 51 (88%) patients in 6 months, and 53 (91%) within a year. Glomerulosclerosis was seen in 20 (35%) patients, fibrosis in 9 (16%), tubular atrophy in 5 (9%), and intimal fibrosis in 3 (5%). The mean±SD serum creatinine in patients within 1-month survival was 1.15±0.19, within 3-month survival was 1.17±0.20, within 6-month survival was 1.21±0.20, within 9-month survival was 1.43±1.28, and within 1-year survival was 1.14±0.22.

Conclusion: Kidney biopsy from live donors can show us a general status of kidney. Serum creatinine is the test of choice for evaluating the grafted kidney function.

## INTRODUCTION

Between 10% and 15% of adult population in the USA suffer from chronic kidney disease (CKD) [1, 2]. This would increase the mortality rate and is an economical burden for health care sectors [3, 4]. One of the best treatments for CKD patients is kidney transplantation. However, a crucial factor to be considered before kidney transplantation from a deceased donor is kidney biopsy from the donated kidney to assess the functionality and efficiency of the transplanted kidney. It is also important in predicting the mid-term and long-term outcomes of the patient [5-9]. Furthermore, the function of the donors’ kidney after transplantation can be evaluated by measuring serum creatinine (Cr) [14-16]. The early outcome of transplantation during the first months of operation was evaluated by measuring serum Cr [16]. Presence of pathological changes in live donors’ kidneys has been shown in previous studies [17]. Considering the increasing number of live donor kidney transplantations in our center, we conducted this study to assess if the presence of histological changes can affect the outcome of recipients.

## MATERIALS AND METHODS

This is a cohort study on patients who referred to Dr. Shari’ati Hospital, Tehran, Iran from April 2010 to April 2011, for renal transplantation, and agreed to participate in this study. Patients younger than 18 years were excluded from the study. 

Samples were taken from the donated kidneys and put in 4% formalin and covered with paraffin. All samples were divided into nine microscopic sections. Four sections were stained with hematoxylin; three with acid schiff; one with Jones method; and the last one with Mallory reagent method. A nephropathologist examined the samples. The pathological changes of interest included tubulitis, severity of acute tubular necrosis (ATN), inflammatory infiltration, glomerulonephritis, hyalinization of arteries, arthritis, fibrosis, tubular atrophy, intimal fibrosis of arteries, and the presence and percentage of glomerulosclerosis. 

All patients had follow-up visit in their 1^st^, 3^rd^, 6^th^, 9^th^, and 12^th^ month of operation to determine if there is any “allograft dysfunction” [14], defined as serum Cr >15% of the baseline value or lack of response to increasing steroid dose [18]. The patients were then categorized into two groups based on their outcome—“successful” and “unsuccessful.” The outcomes were compared based on the biopsy results. 

The immunosuppression regimen we used for our patients included cyclosporine A and prednisolone; cyclosporine A, 5 mg/kg/day, was administered for the 1^st^ three or six months after transplantation; the dose was then tapered down to 12.5 mg. Prednisolone was given at a dosage of 20 mg/day within first three months of operation and tapered down to 10 mg/day thereafter. During the rejection episodes, high dose intravenous prednisolone was administered for five days. 

The participants were signed a written informed consent. Data were analyzed by SPSS^®^ for Windows^®^ ver 18. Correlation between pathological findings and serum Cr was determined by Spearman’s rho. A p value <0.05 was considered statistically significant.

## RESULT

We studied 58 (32 male and 26 female) patients. The mean±SD age of the live donors was 29±7 years. Demographic data of recipients are shown in Table 1. Lab findings of recipients during the follow-up are shown in Table 2. Absence of allograft dysfunction was observed in 55 (95%) patients during the first month, in 51 (88%) in six months, and in 53 (91%) patients in the first year of operation.

**Table 1:** Recipients demographic data

Parameter	Mean±SD	Range
Weight (kg)	62.21± 17.06	22.0–109.0
Height (cm)	158.4±13.0	115.0–183.0
BMI (kg/m^2^)	24.5±5.2	16.6–42.1
Age (yrs)	38.8±14.4	9.0–64.0

The most common observed pathology in biopsies was glomerulosclerosis observed in 20 (35%) patients, followed by fibrosis in 9 (16%), tubular atrophy in 5 (9%), and intimal fibrosis in 3 (5%) patients.

There were significant correlations between presence of allograft dysfunction and fibrosis or intimal fibrosis in the donated kidney (p<0.05), and between allograft dysfunction in the third month and glomerulosclerosis in the donated kidney (p=0.019). 

The presence of intimal fibrosis in biopsies was significantly (p=0.004) associated with a high variance in serum Cr during the 1^st^ month after operation; this is the result of the difference in the mean serum Cr between patients with intimal fibrosis (1.46±0.35 mg/dL) and those without (1.14±0.17 mg/dL). Furthermore, the diversity in the mean serum Cr within one year of operation was high, which is due to the difference between serum Cr in patients with intimal fibrosis (1.46±0.33 mg/dL) and those without (1.31±0.21 mg/dL). 

The mean serum Cr in patients on the first day of operation was 2.3 mg/dL and decreased to 1.1 mg/dL after one year (Table 2). 

**Table 2:** Lab findings in patients after surgery

Parameter	Mean±SD	Range
Urine volume in first 24 hrs (mL)	10,053±3286	3400–20,700
Serum creatinine (mg/dL)		
first day	2.30±0.79	1.10–4.80
at discharge	1.14±0.21	0.80-1.70
1 month	1.15±0.19	0.80–1.80
3 months	1.17±0.20	0.90–1.80
6 months	1.21±0.20	0.90–1.80
9 months	1.43±1.28	0.80–3.20
1 year	1.14±0.22	0.70–1.90

The mean±SD serum Cr in patients who developed allograft dysfunction within the first month of operation (1.32±0.28 mg/dL) was significantly (p=0.001) higher than those without (1.11±0.14 mg/dL). Similar association was found for the third month of operation (mean±SD serum Cr: 1.32±0.22 *vs* 1.11±0.16 mg/dL; p<0.001). 

Both fibrosis and intimal fibrosis in donated kidney are significantly (p=0.002, and p=0.03, respectively) associated with allograft dysfunction during the 1^st^ month of transplantation. There was also significant associations between the 6^th^ month serum Cr>1.5 mg/dL and 6-month survival rate (p=0.009) and 1-year survival rate (p=0.02). Similar associations were observed between 1-month serum Cr>1.5 mg/dL and 9-month survival rate (p<0.001), and between 9-moth serum Cr>1.5 mg /dL and 1-year survival rate (p=0.038).

Allograft dysfunction in the first month of operation was significantly (p=0.002) associated with interstitial fibrosis; graft dysfunction during the third month was significantly (p=0.019) associated with glomerulosclerosis (Table 3).

**Table 3:** Association between glomerulosclerosis and incidence of allograft dysfunction

1^st^ year p value	9^th^ month p value	n	Included biopsies
		20	All cases
0.034	0.503	9	>2 glomeruli
0.030	0.080	4	>4 glomeruli
0.031	0.236	3	>6 glomeruli
0.026	0.048	2	>8 glomeruli

There were only four patients who had both fibrosis and glomerulosclerosis, of whom three developed allograft dysfunction—one after one month, another after nine months, and one after one year. No patient had all the three studied pathological findings.

The mean±SD age of donors with glomerulosclerosis was 30.7±11 years.

## DISCUSSION

Although the mean age of our donors was lower than that reported by other studies [19] (in Schwartz study, for example, the mean age was 47 years, while in our study it was 29), the prevalence of glomerulosclerosis in our series was higher (34%). Kaplan showed that the prevalence of glomerulosclerosis in 55-year-old patients was 0.2%–16.7%; it was 1.5%–23% in 75-year-old patients [20]. In our study, glomerulosclerosis in donated kidney was not associated with graft rejection. Therefore, we should pay attention to the association between glomerulosclerosis and other possible factors that might cause rejection. Absence of allograft dysfunction was observed in 55 (95%) patients during the 1st month, 51 (88%) in 6 months, and in 53 (91%) recipients during the 1st year. The one-year survival rate was 98% in Mosaad report [22], and 100% in both Chamienia report [23] and Hery’s [24]. All of these reports were published in 2011. However, one-year donors’ survival rate was 89% in Hashimura report [25], and 85.6% in Rezaei report, both published in 2004 [26]. Obviously, one-year survival rate has improved for better drug protocols, immunosuppressive drugs, surgical techniques, and the new generation of anti-hypertensive and anti-hyperlipidemic drugs. On the other hand, most foreign studies showed lower rate of graft rejection, probably for relative donors, transfer method, kidney protection and ischemia protocol. Keeping with these reports, Humar showed a graft survival rate of 62% for unrelated living donors—the rate increased to 87% in 1990 [27]. 

During the 1st year of follow-up we had no death. However, a death rate of 9%–30% was reported in the literature due to graft dysfunction [29-30]; the main reason of death was infection [31-34].

The mean serum Cr was 2.3 mg/dL on the 1st day of transplantation; it decreased to 1.1 mg/dL after one year. Therefore, graft function can be closely monitored by serum Cr. We found a significant (p<0.001) association between allograft dysfunction and the mean serum Cr during the 1st month of operation; patients with allograft dysfunction had a mean serum Cr of 1.32 mg/dL and those without allograft dysfunction had a mean serum Cr of 1.11 mg/dL. The same relation existed for the third month of operation too—the mean serum Cr in those with allograft dysfunction (1.32±0.22 mg/dL) was significantly (p<0.001) higher than those without (1.11±0.16 mg/dL).

Alessandro studied 1000 live donors for 28 years and reported a mean serum Cr of 1±0.2 mg/dL before operation; the Cr increased to 1.4±0.3 mg/dL on the discharge day, hence, a rise of 15%. Out of their 1000 live donors, 17% developed post-operative complications [35]. The mean serum Cr on discharging day was 1.14±0.21 mg/dL in our study and 9% of our recipients developed allograft dysfunction within the first year of operation. 

The long-term function of a transplanted kidney is evaluated by measuring serum Cr. In a recent report, it is mentioned that if the serum Cr after six months of transplantation is ≤1.5 mg/dL, the survival rate will be 80%; if it is 2.6–3 mg/dL, the rate will be 55%, and if it exceeds 3 mg/dL the survival rate will be <40% [14]. For recipients who have serum Cr >1.5 mg/dL, an estimated increase in serum Cr of 0.3 mg/dL between 6–12 months of operation is expected [14]. Furthermore, Fitzsimmons, *et al* [15], concluded that serum Cr in 6–12 months of operation can predict the 3-year survival of the grafted kidney. They analyzed 572 patients during six months and 535 during 12 months. The rate of renal failure was 17% in six months and 19.3% in 12 months among patients with serum Cr >1.5 mg/dL; in those with serum Cr >2 mg/dL, the 3-year kidney failure rate was 24.6% in six months and 26.5% in 12 months of operation. The same results were reported by Tejani who studied 686 patients [16].

Several studies showed that taking biopsy from donor’s kidney is not beneficial. However, for lack of enough description of tissue parameters studies and survival rate, the significance of these studies is not questionable [35, 36]. On the other hand, some studies revealed that taking biopsy from donor’s tissue is advantageous [37-39]. For example Soren, *et al* [40], found an association between interstitial fibrosis in biopsies and serum Cr level of 12^th^ month of operation. Leunissen, *et al* [39], found a relation between the severity of tissue complications and serum Cr in the third month of transplantation. Gaber, *et al* [37], in their study found that when glomerulosclerosis is >20% of tissue column, it is clearly associated with 88% of delayed graft function and 38% of graft rejection. The mean serum Cr was 2.6±0.1 mg/dL, however, because the results were come from the analyses of only eight patients with unusual high percentage of glomerulosclerosis, the findings should be interpreted with caution.

Gira, *et al* [37], found a correlation between serum Cr and the 3-month, 6-month, and 1-year kidney survival rate. Based on the findings of Humar and Ishika [41, 42], a serum Cr level >1 mg/dL in six months of operation had a significant correlation with graft failure. In our study, we found a significant correlation between serum Cr level >1.5 mg/dL and 6-month and 1-year graft failure rate. We also observed correlation between serum Cr >1.5 mg/dL in the 1^st^ month and 9-month graft survival rate (p=0.048); serum Cr >1.5 mg/dL in the 9^th^ month and 9-month graft survival (p<0.001); and serum Cr >1.5 mg/dL after one year and 1-year graft survival (p=0.038).

In fact, pre-transplant donor renal biopsy shows the general status of the kidney. Reliability of the biopsy findings was reported in the other studies too [45, 46].

Hass [43] has found correlations between biopsy findings and general problems of the grafted kidney post-operatively (*eg,* atrophy); such correlation was not statistically significant in our study. Ellingsen, *et al* [48], used morphometric counting techniques in evaluating interstitial fibrosis and reported better results than those reported earlier.

In the current study, we found a significant correlation between interstitial fibrosis of the donated kidney and allograft dysfunction, whereas other researchers could not observe such association [49-51].

Presence of glomerulosclerosis in donated kidney though is a good determinant for glomerular grading, there are no consensus statements yet. Some authors mention a range of 5–25 as a reliable grade [52-55]. Glomerulosclerosis has been mentioned as an index of kidney injury in pre-transplant donor renal biopsy [46-47]. In our study, there was a significant correlation between presence of glomerulosclerosis and 3-month graft survival rate. There was also a significant correlation between the presence >2 glomerulosclerosis and 1-year graft survival; the 9-month survival had a correlation with >8 glomerulosclerosis. Therefore, pre-transplant donor renal biopsy has a good predictive value for the grafted kidney survival. Follow-up of these patients for five and 10 years seems necessary to generate more accurate results. Conduction of large cohort studies are thus recommended.
